# Sulfonamide derivative targeting carbonic anhydrase IX as a nuclear imaging probe for colorectal cancer detection *in vivo*

**DOI:** 10.18632/oncotarget.5684

**Published:** 2015-10-05

**Authors:** Siao-Syun Guan, Chun-Chia Cheng, Ai-Sheng Ho, Chia-Chi Wang, Tsai-Yueh Luo, Tse-Zung Liao, Jungshan Chang, Cheng-Tien Wu, Shing-Hwa Liu

**Affiliations:** ^1^ Institute of Nuclear Energy Research, Atomic Energy Council, Taoyuan, Taiwan; ^2^ Institute of Toxicology, College of Medicine, National Taiwan University, Taipei, Taiwan; ^3^ Graduate Institute of Medical Sciences, College of Medicine, Taipei Medical University, Taipei, Taiwan; ^4^ Division of Gastroenterology, Cheng Hsin General Hospital, Taipei, Taiwan; ^5^ Division of Hepatology, Taipei Tzu Chi Hospital, Buddhist Tzu Chi Medical Foundation and School of Medicine, Tzu Chi University, Hualien, Taiwan; ^6^ Department of Medical Research, China Medical University Hospital, China Medical University, Taichung, Taiwan; ^7^ Department of Pediatrics, National Taiwan University Hospital, Taipei, Taiwan

**Keywords:** colorectal cancer, carbonic anhydrase IX, sulfonamide, radioisotope-labeled

## Abstract

Hypoxic microenvironment is a common situation in solid tumors. Carbonic anhydrase IX (CA9) is one of the reliable cellular biomarkers of hypoxia. The role of CA9 in colorectal cancer (CRC) remains to be clarified. CA9 inhibitor such as sulfonamides is known to block CA9 activation and reduce tumor growth consequently. Here, we aimed to investigate the CA9 expression in serum and tumor from different stages of CRC patients and utilize sulfonamide derivative with indium-111 labeling as a probe for CRC nuclear imaging detection *in vivo*. The serum CA9 was correlated with the tumor CA9 levels in different stages of CRC patients. Hypoxia increased cell viability and CA9 expression in colorectal cancer HCT-15 cells. Sulfonamide derivative 5-(2-aminoethyl)thiophene-2-sulfonamide (ATS) could bind with CA9 *in vitro* under hypoxia. Moreover, tumor tissues in HCT-15-induced xenograft mice possessed higher hypoxic fluorescence signal as compared with other organs. We also found that the radioisotope signal of indium-111 labeled ATS, which was utilized for CRC detection in HCT-15-induced xenograft mice, was markedly enhanced in tumors as compared with non-ATS control. Taken together, these findings suggest that CA9 is a potential hypoxic CRC biomarker and measurement of serum CA9 can be as a potential tool for diagnosing CA9 expressions in CRC clinical practice. The radioisotope-labeled sulfonamide derivative (ATS) may be useful to apply in CRC patients for nuclear medicine imaging.

## INTRODUCTION

Colorectal cancer (CRC) is one of most common malignancy cancers worldwide [1]. Epidemiological studies have shown that the incidence and mortality of colorectal cancer is increasing over the past several decades [2, 3]. The clinicopathological characteristics of CRC patients are well known, including the factors of age and tumor site [4–6]. In addition, the higher mortality of CRC is due to the disease that is frequently diagnosed in the advanced stage without reliable diagnosis. Thus, accurate detection of cancerous lesions can provide more effective surgical and pharmacological therapies for decreasing in the mortality of CRC [7]. Colonoscopies are considered the most preferred tool for CRC screening in clinical; however, they are invasive and present a small but significant risk for perforations and limitations in making an accurate diagnosis due to sampling errors or personal skill [8, 9]. The development of high sensitivity and specificity diagnostic methods is urgent and important.

Hypoxia (low oxygen tension) is a biologically important phenomenon with strong effects on cancer progression and tumor phenotype [10]. The hypoxic tumor cells rapidly outgrow their blood supply leading to portions of the tumor with regions where the oxygen concentration is markedly lower (typically ≦1% of overall oxygen content) than in healthy tissues, and accompany a decrease in extracellular pH (~ pH 6.5) in the tumor microenvironment [11, 12]. In order to survive in the acidic microenvironment, these tumor cells have to maintain an intracellular pH near physiological levels (pH 7.2–7.4) [13]. Carbonic anhydrase IX (CA9), a zinc metallo-enzyme, is one of 15 human isoforms of carbonic anhydrase family, which catalyzes the reversible hydration of carbon dioxide to bicarbonate and a proton [14, 15]. CA9 has been observed to be directly linked to an increase of hypoxia inducible factor (HIF)-1, and to correlate to cell survival, proliferation, migration, growth, adhesion, pH value, and cell-signaling pathways [16, 17]. Previous studies have shown that CA9 is predominantly associated with and high expressions in many human tumor types, including breast [18], lung [19], kidney [20], cervix uteri [21], oral cavity [22], brain [23], pancreas [24], and gastric epithelium [25]; but its decreased expression may be associated with human gastric carcinogenesis [26]. Hence, more attention is focused on the CA9 that is being pursued as a biomarker for development of specific systemic therapies and diagnostic imaging probe [27, 28].

Carbonic anhydrase inhibitors have been extensively studied and their inhibition mechanisms were well-established for carbonic anhydrase-correlated imaging and therapy [29]. Sulfonamide-based compounds (sulphonamides, sulphanilamides, sulphamates and their derivatives) are CA9 small molecular inhibitors, which inhibit CA9 by coordinating to the zinc ion within the active site with μM to nM Ki inhibition [30, 31]. They are the most robust class of CA9 inhibitors due to their high affinity, availability, and ease of chemical manipulation [32]. Fluorescent dye-conjugated sulfonamides have been developed and revealed the binding affinities to CA9 [33]. The fluorescent sulfonamides for measuring CA9 expressions have been used in renal and breast tumor xenografts [34–36]. In addition, the near-infrared (NIR) fluorescent labeled sulfonamide derivatives were synthesized for imaging hypoxia-induced CA9 expressions in tumor cells *in vitro, ex vivo*, and *in vivo* [37]. However, the specific efficiency of sulfonamides on CA9-overexpressed CRC remains to be clarified.

Nuclear imaging is known as a common clinical-used diagnostic tool in several cancers for providing a prominent proportion of information in surgical management, radiation planning, chemotherapeutic assessment, and follow-up evaluation of patients [38]. The nuclear imaging with cancer-specific probes such as monoclonal antibodies, peptides, nanoparticles and other specific small molecular compounds are potentiality for tumor diagnosis [39–43]. Even though technetium-99m labeled sulfonamide derivative has been developed for CRC detection, the results were undesirable [44]. Therefore, in the present study, we aimed to investigate whether (1) serum CA9 (sCA9) levels are correlated with tumor tissue CA9 levels in clinical CRC patients and can as a CRC reliable biomarker; (2) CA9 can as a biomarker for hypoxic tumor diagnosis; (3) radiolabeled sulfonamide derivative can bind to CA9-overexpressed CRC tumors.

## RESULTS

### The levels of sCA9 and tumor tissue of CRC patients

**Figure 1:**
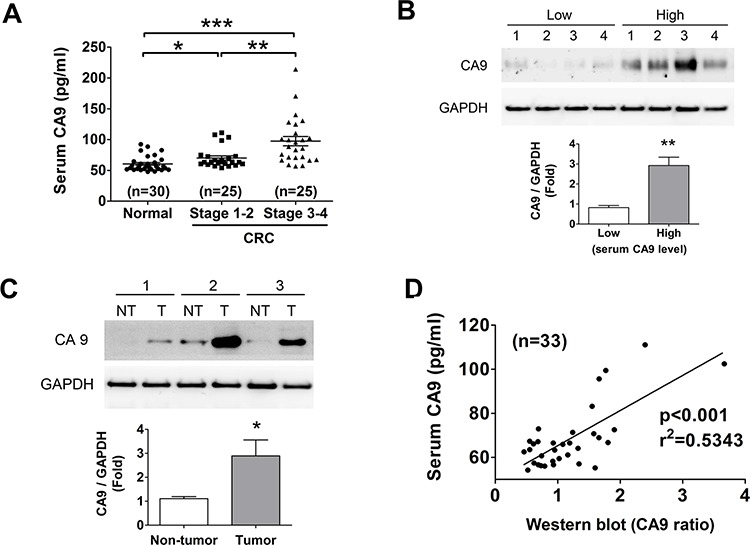
Evaluation of CA9 as a CRC biomarker **A.** The changes of serum CA9 (sCA9) levels in normal subject and CRC patients with stage 1–2 and stage 3–4 detected using an ELISA assay. *P < 0.05, **P < 0.01, ***P < 0.001. **B.** CA9 protein expression in CRC tissues responding the sCA9 levels. The tissues were chosen from four high and four low sCA9 levels of CRC patients for Western blotting. Protein levels were quantified by densitometry and normalized by GAPDH levels. The data are showed as means ± SEM (*n* = 4). **P < 0.01, versus low sCA9 levels of CRC patients. **C.** The protein expressions of CA9 in tumors and non-tumors from the individual CRC patients with high sCA9 levels. Protein levels were quantified by densitometry and normalized by GAPDH levels. The data are presented as means ± SEM (*n* = 3). *P < 0.05, versus non-tumor. **D.** The sCA9 levels were correlated with tumor tissue CA9 protein expressions in CRC patients. *p < 0.001, versus sCA9.

In order to evaluate CA9 as a CRC biomarker, we performed ELISA assay to observe the serum CA9 (sCA9) levels of CRC patients as compared to healthy volunteers. There were about 32% (8/25) of early stage and 44% (11/25) of late stage CRC patients displaying high-level sCA9 more than the average of sCA9 concentration in disease group, and about 76% (early stage:17/25, late stage: 21/25) of CRC patients revealing high-level sCA9 more than the average of sCA9 levels in control group (Figure 1A). The clinical characteristics of CRC patients and healthy volunteers were shown in Table 1. To detect the correlation between sCA9 and tissue CA9 in the individual CRC patients, protein levels of CA9 in tumor tissues from patients with low- and high-level sCA9 were measured. As shown in Figure 1B, the protein expressions of CA9 in tumors were higher in patients with high-level sCA9 than in patients with low-level sCA9. Moreover, the CA9 expressions were also analyzed in tumor and non-tumor tissues from patients with high-level sCA9. As shown in Figure 1C, higher CA9 expressions were observed in tumor tissues than in non-tumor tissues. The sCA9 levels were correlated with tissue CA9 expressions in CRC patients (*p* < 0.05, Figure 1D), considering that detection of sCA9 may be used to reflect the levels of CA9 in tumors as a reference during cancer therapy.

**Table 1:** The clinical characteristics of normal volunteers and CRC patients

	Normal volunteers (*n* = 30)	CRC patients (stage 1–2, *n* = 25)	CRC patients (stage 3–4, *n* = 25)
Age (years)	53.63 ± 10.97	62.44 ± 10.02	64.96 ± 13.17
Gender			
Male	15	14	12
Female	15	11	13
Body weight	63.76 ± 12.21	63.08 ± 11.93	59.55 ± 11.50
BMI (kg/m^2^)	23.67 ± 3.69	24.68 ± 3.58	23.84 ± 2.77
Serum CEA (ng/dL)			
<1	15	0	0
1–5	15	19	10
>5	0	6	15
Smoker	1	4	4
Non-smoker	29	21	21
Moderate or heavy drinker	3	1	0
Light or non-drinker	27	24	25

Abbreviations: BMI, body mass index; CEA, carcinoembryonic antigen.

### Histological and immunohistochemical analysis of clinical specimens of CRC

**Figure 2:**
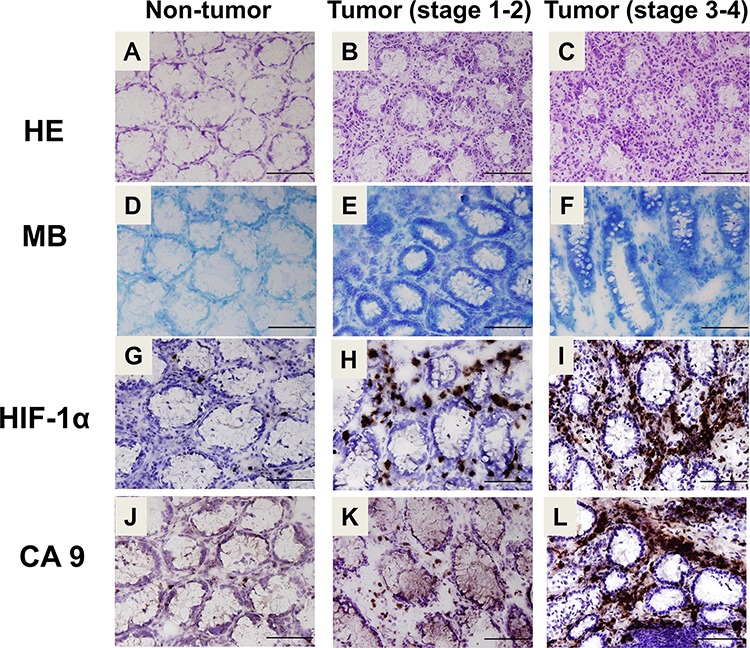
Histological and immunohistochemical analysis for hypoxia and CA9 expression in human colon cancer **A–C.** Colorectal sections of CRC patients were stained with hematoxylin and eosin (A: non-tumor, B: stage 1–2, C, stage 3–4). Top panel: × 400, scale bar: 100 μm. **D–F.** Histopathological confirmation of non-tumor and cancerous tumor tissues (D: non-tumor, E: stage 1–2, F: stage 3–4) of each colorectal specimen was carried out by staining with methylene blue. The immunohistochemical staining for HIF-1α **G–I.** and CA9 **J–L.** was performed on the colorectal sections of human CRC patients (G and J: non-tumor, H and K: stage 1–2, I and L: stage 3–4). Top panel: 400x, scale bar: 100 μm. HE: hematoxylin and eosin. MB: methylene blue. HIF-1α: hypoxia-inducible factor-1.

The histology of normal and cancer colon tissue were shown in Figure 2A–2C. The results exhibited strong staining of nuclei of (proliferating) epithelial cells in the cancer colon mucosa and stroma as compared with normal tissue. In order to confirm the cancerous tumor and non-tumor tissue, methylene blue stain was performed to observe in CRC patients colon tissue (Figure 2D–2F). To investigate tissue hypoxia condition in the different CRC stages, we analyzed HIF-1α, which have been proposed as a potential cell hypoxic biomarker for cell- and tissue-based detection by using immunohistochemical assay [45]. The immunohistochemical staining was conducted for CA9 and HIF-1α to verify their protein expressions within the normal and colon tumor tissues. HIF-1α exhibited strong staining in the colon cancerous tissues (Figure 2H and 2I). Within normal tissue, a majority of epithelial cells stained negative for HIF-1α (Figure 2G). Moreover, CA9 also exhibited strong staining within the tumor tissues (Figure 2K and 2L). Little to no staining was observed for CA9 in human normal colon tissues (Figure 2J). Interestingly, the expressions of CA9 and HIF-1α in late stage of CRC tissue were higher than in early stage of CRC tissue.

### Hypoxia condition induced cells proliferation and CA9 overexpression in human CRC cells

**Figure 3:**
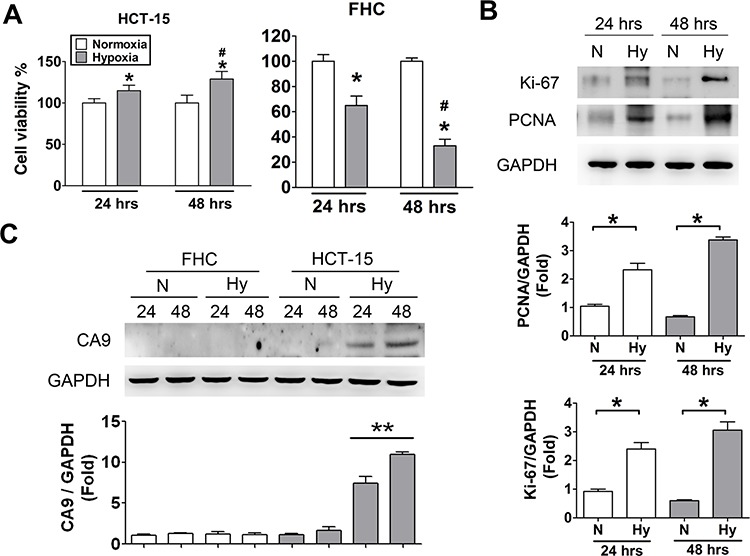
Hypoxia induced cell proliferation and CA9 overexpression in human CRC cells **A.** Cell viability assay. HCT-15 and FHC cells were incubated at normoxic and hypoxic condition for 24 and 48 h. Data were presented as means ± SEM (*n* = 5). *P < 0.05, versus normoxia; #*P* < 0.05, versus hypoxia 24 h. **B.** Cell proliferation detected in hypoxic HCT-15 cells. After incubating at hypoxia and normoxia for 24 and 48 h, the protein expressions of Ki-67 and PCNA in HCT-15 cells were determined by Western blotting. Protein levels were quantified by densitometry and normalized by GAPDH levels. The data are showed as means ± SEM (*n* ≥ 3), *P < 0.05. N: normoxia, Hy: hypoxia. **C.** The protein expressions of CA9 in hypoxic tumor and normal cells. The HCT-15 and FHC cells were incubated at hypoxia for 24 and 48 h to measure CA9 levels determined by Western blotting. Protein levels were quantified by densitometry and normalized by GAPDH levels. The data are exhibited as means ± SEM (*n* ≥ 4). **P < 0.01, versus normoxia 24 h. N: normoxia, Hy: hypoxia.

We indicated that the hypoxia levels in CRC tumor tissues were higher than non-tumor tissues (Figure 2). In order to estimate the effects of hypoxia, the cell viability were measured in colorectal tumor cells (HCT-15) and human normal colon cells (FHC). As shown in Figure 3A, the cellular viability was significantly increased in HCT-15 under hypoxia condition for 24 and 48 hours as compared with normoxic condition. However, the cellular viability was markedly decreased in human normal colon cells (FHC) under hypoxia condition. To analyze the effect of hypoxia on cellular proliferation in HCT-15 cells, the cellular proliferated markers, Ki-67 and PCNA, were detected by immunoblotting assay. The protein expressions of Ki-67 and PCNA were predominantly enhanced in hypoxia condition for 24 and 48 hours (Figure 3B). We further observed the expression of CA9 in HCT-15 and FHC cells under normoxic and hypoxic condition. As shown in Figure 3C, the expression of CA9 was remarkably up-regulated in HCT-15 under hypoxia, but there was no effect in FHC cells under normoxic or hypoxic condition. In addition, the expression of CA9 under hypoxia for 48 hours was higher than hypoxia for 24 hours.

### Hypoxia and CA9 expression in HCT-15-induced xenograft mice

**Figure 4:**
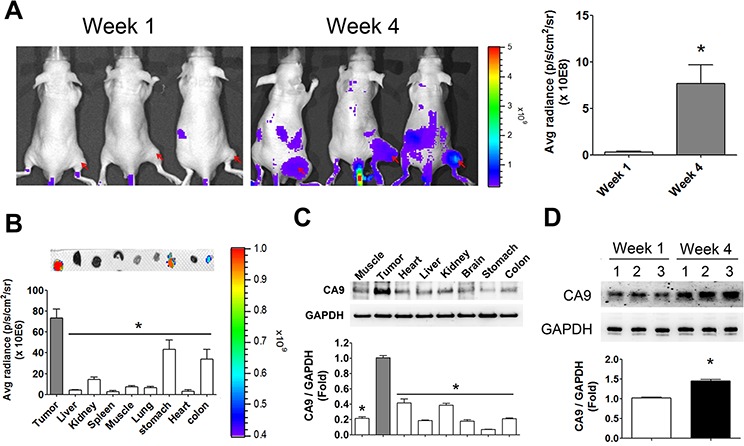
Hypoxia imaging in the tumors and organs of HCT-15-induced xenograft mice **A.** The hypoxic imaging detection in 1-week and 4-week HCT-15 xenograft mouse model. The hypoxic antibodies labeled with fluorescent were injected into mice for 24 h and analyzed by *in vivo* imaging system (IVIS). The arrows indicated the tumor site of HCT-15-induced xenografts for imaging quantification. Data are presented as means ± SEM (*n* ≥ 3). *P < 0.05, versus week 1 group. **B.** The hypoxia-distributed imaging in various organs of HCT-15 xenograft mice. After whole animals imaging assay, HCT-15 xenograft mice were sacrificed and acquired tumor, liver, kidney, spleen, muscle, lung, stomach, heart, and colon for fluorescent imaging assay by IVIS. The top panel showed the imaging of hypoxia in various organs. Button panel displayed quantitation of fluorescent signals. Data are presented as means ± SEM (*n* ≥ 3). *P < 0.05, versus tumor. **C.** The protein expressions of CA9 in various organs of HCT-15 xenograft mice. Following organs fluorescent imaging assay, all organs were homogenized for Western blotting. Protein levels were quantified by densitometry and normalized by GAPDH levels. All data are showed as means ± SEM (*n* = 3). *P < 0.05, versus tumor. **D.** The expressions of CA9 in tumor site of HCT-15-induced xenograft mice. Following organs fluorescent imaging assay, the tumors of 1-week and 4-week HCT-15-induced xenograft mice were homogenized for western blotting. Protein levels were quantified by densitometry and normalized by GAPDH levels. All data are exhibited as means ± SEM (*n* = 3). *P < 0.05, versus 1-week HCT-15-induced xenograft mice.

We next observed the tissue hypoxic imaging in HCT-15-induced xenografts in mice. The fluorescence signals were markedly enhanced in the 4-week HCT-15-induced xenograft mice as compared with 1-week HCT-15-induced xenograft mice (Figure 4A). The distributions of hypoxia in several organs of 4-week HCT-15-induced xenograft mice were also estimated. Tumor tissue possessed higher fluorescence signals as compared with other organs (Figure 4B). The protein expression of CA9 was also higher in tumor tissue than in other organs (Figure 4C). The CA9 levels were up-regulated predominantly in 4-week HCT-15-induced xenografts as compared with in 1-week HCT-15-induced xenografts, which was consistent with the results of fluorescence imaging (Figure 4D).

### Sulfonamide derivative interacted with CA9 protein and decreased HCT-15 cell viability under hypoxia

**Figure 5:**
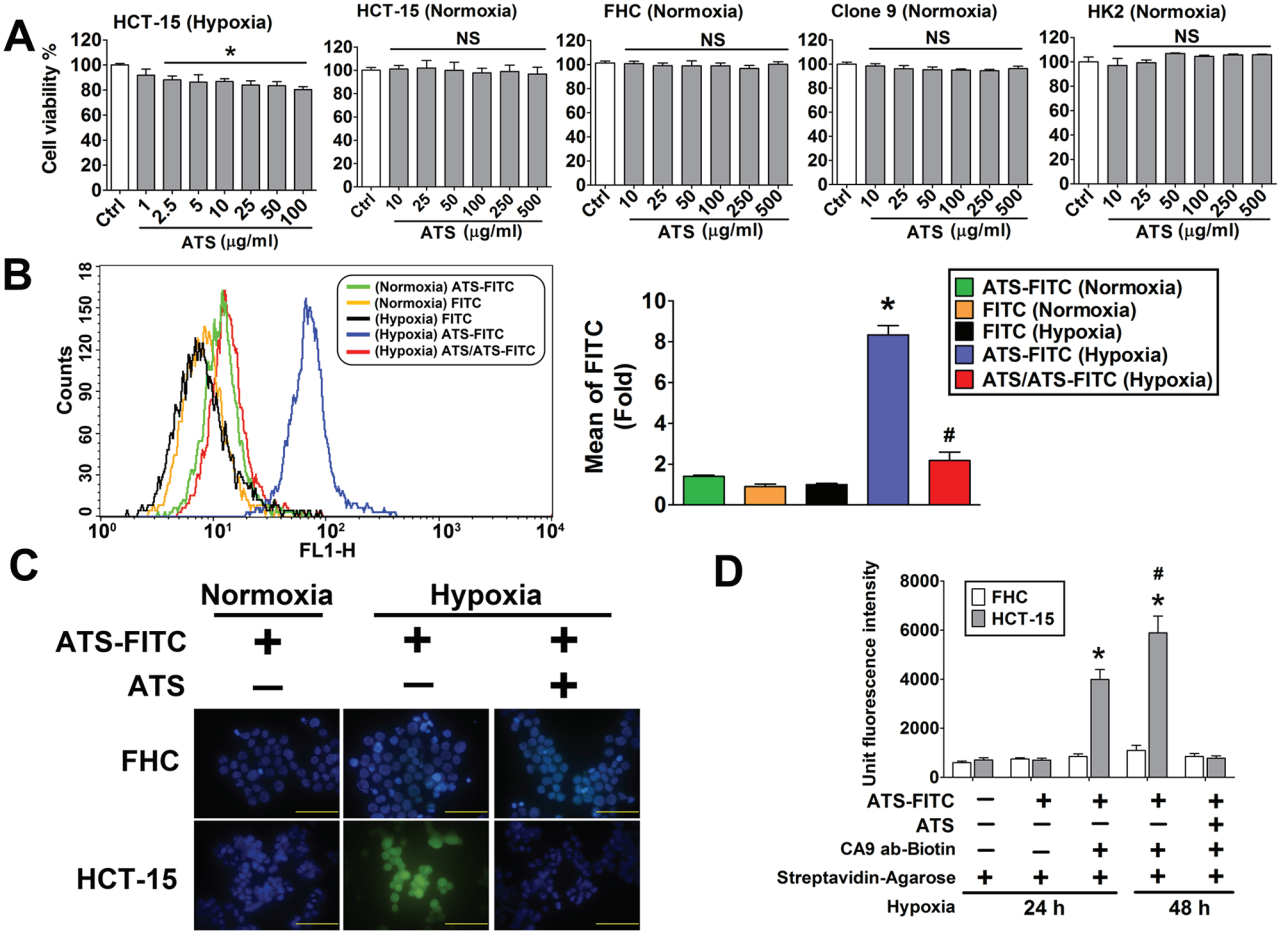
Effects of ATS on HCT-15 cell viability and interaction with the CA9 under hypoxic condition **A.** The effect of ATS on cell viability. HCT-15, FHC, Clone 9 and HK2 cells were treated with ATS (1–500 μg/ml) for 24 h. Data were presented as means ± SEM (*n* = 5). *P < 0.05, versus control; NS: non-significant. **B.** ATS binding assay were determined by flow cytometric assay in HCT-15 cells. The HCT-15 cells were treated with FITC-labeled ATS (ATS-FITC, 10 μg/ml) or FITC (10 μg/ml) under normoxia or hypoxia for 24 h. In competitive group, HCT-15 cells were pre-treated with ATS (10 μg/ml) for 1 hour and then treated with FITC-labeled ATS (ATS-FITC, 10 μg/ml) for 24 h under hypoxia. Data are presented as means ± SEM (*n* = 3). *P < 0.05, versus FITC (Hypoxia); #*P* < 0.05, versus ATS-FITC (Hypoxia). **C.** ATS binding assay was observed by fluorescence microscopy in HCT-15 and FHC cells. The HCT-15 and FHC cells were treated with FITC-labeled ATS (ATS-FITC, 10 μg/ml) under normoxia and hypoxia for 24 h. The ATS (10 μg/ml) was pretreated for competitive inhibition assay. Magnification: 1000x, scale bar: 50 μm. **D.** ATS and CA9 interaction assay. After hypoxia 24 and 48 h, HCT-15 and FHC cells (1 × 10^6^ cells) were treated with fluorescent labeled ATS (ATS-FITC, 10 μg/ml) for 4 h at 4°C. The supernatant of lysates was incubated with or without biotinylated-CA9 polyclonal antibody (2 μg/ml) in the presence of streptavidin agarose beads at 4°C overnight. The fluorescent signaling of immunoprecipitates was detected by using ELISA reader. In competitive group, The ATS (10 μg/ml) was pretreated for competitive inhibition assay. Data were presented as means ± SEM (*n* = 3). *P < 0.05, versus group 2; #*P* < 0.05, versus group 3.

Sulfonamide derivative, 5-(2-aminoethyl)thiophene-2-sulfonamide (ATS) (2.5–100 μg/ml), decreased cell viability of HCT-15 cells under hypoxic condition (Figure 5A). However, there was no cytotoxicity in normoxic HCT-15 and other normal cells, including FHC, rat normal liver cells (Clone 9) and human normal kidney cells (HK2) (Figure 5A). Furthermore, the flow cytometry analysis was performed to measure the ATS specific function in hypoxic HCT-15 cells. As shown in Figure 5B, fluorescein isothiocyanate (FITC)-labeled ATS (ATS-FITC) was markedly bound to hypoxic HCT-15 cells. The binding level of ATS-FITC was predominantly decreased in unlabeled ATS-pretreated HCT-15 (Figure 5B). To confirm the binding ability of ATS-FITC in tumor and normal cells, immunofluorescence staining was performed to analyze in HCT-15 and FHC cells under hypoxic and normoxic condition. The results revealed that fluorescence imaging was prominently raised as compared to normoxic HCT-15 cells (Figure 5C). The fluorescence signals were significantly inhibited in unlabeled ATS-pretreated HCT-15 cells. In addition, the fluorescence imaging was also not detected in hypoxic and ATS-pretreated FHC cells. We further demonstrated that ATS could bind to HCT-15 cells via interacting with CA9 by using immunoprecipitation assay (Figure 5D).

### The nuclear imaging agent of ATS-DTPA-^111^In for CRC tumor detection *in vivo*

**Figure 6:**
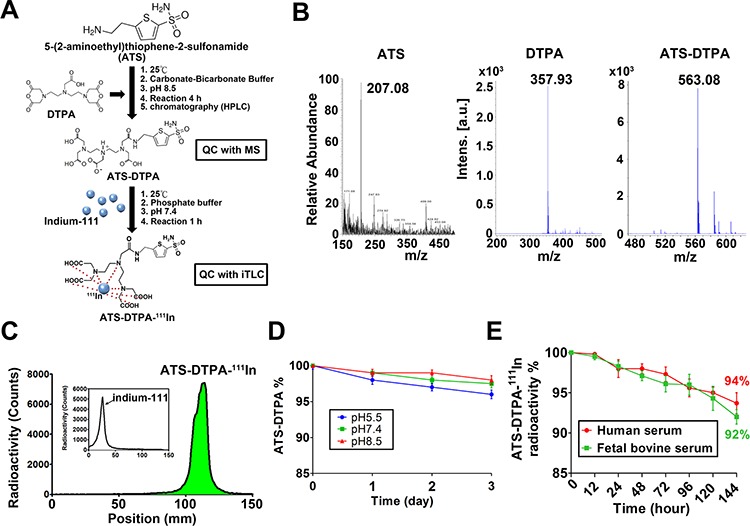
The ATS-DTPA-^111^In synthesis and stability assay **A.** The flowchart of ATS-DTPA-^111^In synthesis was illustrated, where ATS was linked with DTPA for ^111^In labeling. **B.** The molecular weight of ATS, DTPA and ATS-DTPA-^111^In was determined using mass spectrometry. The molecular weight of ATS, DTPA, and ATS-DTPA was measured as 207.08, 357.93, and 563.08 Da, respectively. **C.** The labeling efficiency of ATS-DTPA-^111^In. The conjugation ATS-DTPA was labeled with indium-111 in phosphate buffered saline for 1 h. Instant thin layer chromatography (ITLC) was performed to analyze the radio-labeling efficiency. The radio-labeling efficiency of ATS-DTPA-^111^In was more than 98%. The inserted figure indicated the free indium-111 position in the ITLC. **D.** *In vitro* stability of ATS-DTPA in various pH buffers. The levels of ATS-DTPA were observed at different buffer conditions (pH 5.5, 7.4, and 8.5) for 3 days at 37°C and detected by HPLC. Data are presented as means ± SEM (*n* = 5). *p < 0.05, versus day 0. **E.** *In vitro* stability of ATS-DTPA-^111^In in human and fetal bovine serum. The free indium-111 from ATS-DTPA-^111^In was estimated at human and fetal bovine serum for 144 hours at 37°C and measured by ITLC. The radioactivity of ATS-DTPA-^111^In was preserved more than 94% and 92% at 144 hours in human and fetal bovine serum, respectively. Data are presented as means ± SEM (*n* = 5).

Next, we tried to synthesize the ATS-conjugated diethylene triamine pentaacetic acid (DTPA)-^111^In (ATS-DTPA-^111^In) complex to diagnose the CRC in a xenograft mouse model. DTPA is known to specifically trap the indium-111 radioisotope. The construct of the sulfonamide-based nuclear imaging agent and synthesis flow chart were illustrated in Figure 6A. After synthesis and purification, the molecular weight of ATS-DTPA compounds was analyzed to determine the successful rate of conjugations. DTPA was successfully conjugated with ATS determined by mass spectrometry assay (Figure 6B). To ensure the labeling efficacy of indium-111 onto the ATS-DTPA, instant thin layer chromatography was performed to separate indium-111 and indium-111 labeled ATS-DTPA (ATS-DTPA-^111^In) in 15 cm membrane electrophoresis and estimated the radioactive signals by using a gamma counter. The ATS-DTPA labeling efficacy with indium-111 was about 98% (Figure 6C), demonstrating that the sulfonamide-based nuclear imaging agent was synthesized with stable conjugation in phosphate buffered saline. To measure the stability of ATS-DTPA, the concentrations of ATS-DTPA in various pH conditions (pH = 5.5, 7.4, and 8.5) were analyzed. The results exhibited that the ATS-DTPA levels were no difference for 3 days in different pH condition (Figure 6D). In addition, the stability of ATS-DTPA-^111^In in human and fetal bovine serum was observed in a 144-hours period. The results displayed that the stability of ATS-DTPA-^111^In was preserved more than 94% and 92% for 144 hours in human and fetal bovine serum, respectively (Figure 6E).

**Figure 7:**
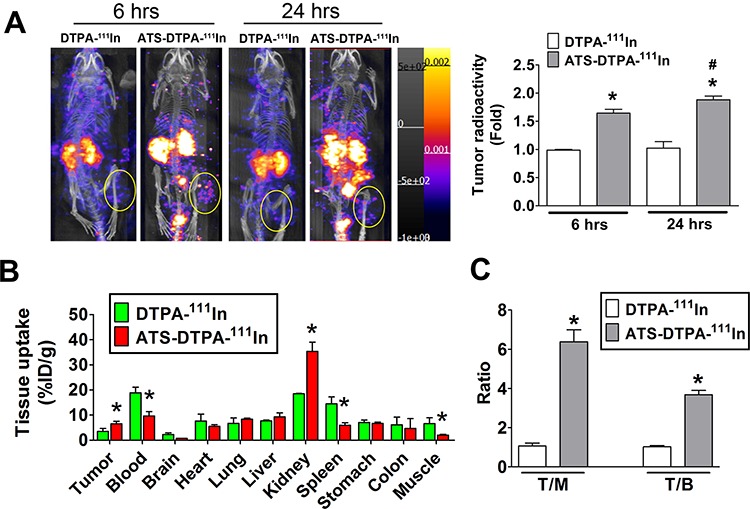
The nuclear imaging agent of ATS-DTPA-^111^In for CRC tumor detection *in vivo* **A.** The CRC tumor nuclear imaging analysis in HCT-15-induced xenograft model. The ATS-DTPA-^111^In was injected into mice via intravenous for 6 and 24 h and measured by using nanoSPECT/CT. DTPA-^111^In was a control reagent. The oval-shaped labeling displayed the tumor site of HCT-15-induced xenograft mice for imaging quantification. Data are presented as means ± SEM (*n* ≥ 3). *P < 0.05, versus DTPA-^111^In group. #*P* < 0.05, versus ATS-DTPA-^111^In 6 h group. **B.** Tissue bio-distribution of ATS-DTPA-^111^In in HCT-15-induced xenograft mice. After 24 h ATS-DTPA-^111^In and DTPA-^111^In intravenous injection, HCT-15-induced xenograft mice were sacrificed and acquired tumor, blood, brain, heart, lung, liver, kidney, spleen, muscle, stomach, and colon for radioactivity assay determined by a gamma counter. Values are expressed as the percentage of injected dose per gram organ (% ID/g). Data are presented as means ± SEM (*n* ≥ 3). *P < 0.05, versus DTPA-^111^In tumor 6 h group. **C.** Comparison of tumor-to-muscle (T/M) and tumor-to-blood (T/B) ratios among DTPA-^111^In and ATS-DTPA-^111^In injected mice at 24 h after injection. Data are expressed as means ± SEM (*n* ≥ 3). *P < 0.05, versus DTPA-^111^In group.

In order to detect the imaging efficiency of ATS-DTPA-^111^In, the CRC tumor imaging was measured in the 4-week HCT-15-induced xenograft mice. After 6 and 24 hours imaging agent injection, the radionuclide signals were significantly increased in ATS-DTPA-^111^In-treated mice as compared with control DTPA-^111^In-treated mice (Figure 7A). Then, the 24 hours distributions of ATS-DTPA-^111^In in several organs of 4-week HCT-15-induced xenografts were also estimated. Tumor tissue possessed higher ATS-DTPA-^111^In signals as compared with DTPA-^111^In groups (Figure 7B). Moreover, the bio-distribution of ATS-DTPA-^111^In and DTPA-^111^In were also markedly difference in blood, kidney, spleen and muscle (Figure 7B). We further detected the ratios of tumor-to-muscle (T/M) and tumor-to-blood (T/B) in ATS-DTPA-^111^In and DTPA-^111^In injected HCT-15-induced xenografts. The ATS-DTPA-^111^In injected mice showed higher ratio in T/M and T/B compared with DTPA-^111^In injected mice (Figure 7C), indicating that ATS-DTPA-^111^In could penetrate into CRC tumor hypoxic tissues and consequently improve the imaging efficacy.

### Toxicity evaluation for ATS-DTPA

**Figure 8:**
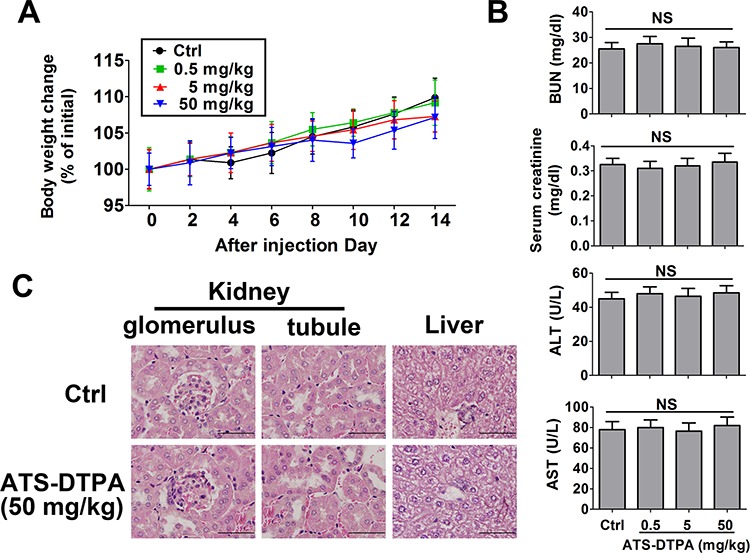
Toxicity evaluation of ATS-DTPA in mice for 14 days BALB/c mice were divided into 4 groups (*n* = 10 in each group). Group I was control group and received only vehicle (PBS). Group II, III, and IV were treated with ATS-DTPA at doses of 0.5, 5, 50 mg/kg, respectively. Mice were administered with a signal dose intravenous injection of vehicle or ATS-DTPA. **A.** The body weight changes (% of initial). After ATS-DTPA treated, the body weight changes (% of initial) in all group were determined daily for 14 days. **B.** Serum BUN, creatinine, ALT, and AST assays. BUN: blood urea nitrogen. ALT: alanine aminotransferase. AST: aspartate aminotransferase. NS: non-significant. **C.** Histological analysis of kidney and liver. The sections of liver and kidney were stained with hematoxylin and eosin. Original magnification 1000x, scale bar: 50 μm.

We next tried to do a preliminary toxicity evaluation for ATS-DTPA in mice after signal dose intravenous injection. There were no deaths for all experimental groups within 14 days observation. There were no differences in body weight change among four experimental groups (Figure 8A). The indicators of renal function (BUN and creatinine) and liver function (alanine aminotransferase and aspartate aminotransferase) were no difference between control and ATS-DTPA-treated groups (Figure 8B). Moreover, histological evaluation of liver and kidney from control and ATS-DTPA (50 mg/kg)-treated mice revealed no imprint of toxicological signature in these organs (Figure 8C).

## DISCUSSION

In this study, we identified that CA9 levels in serum are correlated with tumor tissue CA9 levels in clinical CRC patients and CA9 can as a biomarker for hypoxic tumor diagnosis. We also found that sCA9 and tumor CA9 levels in late stage of CRC patients were higher than in early stage of CRC patients. In addition, hypoxia could cause cell proliferation and increase CA9 expressions in cultured CRC cells. The sulfonamide derivative ATS directly and specifically reacted with CA9 in hypoxic CRC cells. We also provided a novel method that sulfonamide conjugated with DTPA followed ^111^-In labeling. The ATS-DTPA-^111^In was successfully captured by CRC tumor imaging *in vivo*. In addition, the serum biochemistry values and histology staining indicated that ATS-DTPA compounds were no toxic effects in blood circulation of animals. These findings presume that ATS effectively binds to CA9 overexpressed CRC cells and ATS-DTPA-^111^In, providing a new nuclear imaging reagent for CA9-overxpressed CRC tumor detection.

The non-invasive NIR fluorescence imaging is known as a clinical application for detection of human deep organs. However, the limitation of tissue penetration let it to be unrealistic. It has been suggested to require considerable improvement of sensitivity of the fluorescence imager and quantum efficiency of fluorophores [45]. In addition, even though Akurathi et al. developed technetium-99m (Tc-99m)-labeled sulfonamide for CRC detection [46], the results were undesirable. They only collected 0–4 h drug biodistribution data, but not imaging data. The bio-distribution of [99mTc]-sulfonamide in CA9 expressing tumor bearing mice at 0.5, 1, 2 and 4 h (i.p.) showed low tumor accumulation (< 0.1% ID). We presumed that the lower accumulated radiolabeled drug resulted in image collection with difficulty. In the present study, the result of bio-distribution of In-^111^-labeled sulfonamide showed tumor hypoxia accumulation more than 5% ID after 24 h i.v. injection (Figure 7B). Therefore, we consider that isotope labeling is better than NIR fluorescent labeling for hypoxia CRC detection in clinical and In-^111^ is more beneficial for sulfonamide labeling compared with Tc-99m.

It has been recognized for several decades that there was a correlation between tumor hypoxia and resistance to chemotherapy and radiotherapy [47–50]. Though surgery of CRC is the foundation of curative treatment in more than 90% of cases, chemotherapy is maintained after tumor resection due to the high risk of recurrence in advanced cancers [51]. In addition to a few CRC cases, radiotherapy is a major method for head-and-neck cancers treatment in clinical [52]. Therefore, tumor hypoxia detection could provide physician to determine the optimal drug usage and decreased time redundancy in tumor treatment. CA9 is presumed as one of the most prominent markers of tumor hypoxia with potential to serve as a diagnostic biomarker, prognostic indicator as well as tumor therapeutic target in many cancers [53, 54]. In the present study, we demonstrated that CA9 was overexpressed in hypoxic CRC tumor as compared with normoxia condition. CA9 may be considered to be as a biomarker for hypoxic CRC tumor diagnosis.

The colonoscopy and histological examination of biopsy specimens are considered the gold standard for diagnosis of CRC in clinical practice [55]. However, the poor resolution of endoscopy and sampling error in small CRC nodules and interpersonal variations may decrease the accuracy of tumor analysis. In addition, immunohistochemistry (IHC) is the most generally methods to estimate the protein status in cancerous samples. Even though IHC exhibited high standardization and semi-quantitative assay, it has numerous limitations, including technical operation and illustration, which have been found to impact on the reproducibility and accuracy of results. Moreover, the complicated and time-consuming processes lead to undesirable screening in a lot of clinical samples. The high throughput serum biomarkers screening for CRC provides results acquired quickly and interpreted easily to doctor for deciding tumor therapeutic method. Currently, FDA has approved human serum detection kits to determine the status of CA9 expressions. High levels of CA9 in plasma and serum have been measured to be raised in the patients with breast, lung, gastric and cervical cancer [19, 56–58]. In contrast to tumor tissue CA9 protein analysis, serum CA9 level examination in CRC patients is relatively rare. In the present study, we also observed that the average serum CA9 levels were remarkably increased in CRC patients, which were correlated with tumor tissue CA9 expressions. In addition, the serum CA9 levels in early stage of CRC patients were significantly higher than normal control group. These results implied that serum CA9 levels determined by ELISA assay might be a profitable and invasive tool for diagnosing CA9 expressions in CRC patients.

In the study for G250 monoclonal antibody, it has been displayed excellent specificity for CA9 in clear cell renal cell carcinoma (CCRCC) analysis [59]. Moreover, The M75 was another highly specific antibody for CA9 [60], which recognized the extracellular proteoglycan-like domain of CA9 and widely used for western blotting, immunoprecipitation and IHC in human tumor tissues. Compared to CA9 specific monoclonal antibody, the CA9 specific small molecule-based probes provided some advantages for tumor imaging. Sulfonamides has been demonstrated to only bind to active CA9, indicating that the active site of CA9 is only available for sulfonamides binding in several cell lines during hypoxia [33, 61]. Nevertheless, the transmembrane CA9 proteins are very stable (with a half-life of about 40 hours) for a relatively long time after reoxygenation. Therefore, sulfonamides can distinguish cells that are currently in hypoxic conditions from those that are previously hypoxic, while CA9 specific antibodies cannot do it. In addition, sulfonamides are relatively easy to make and low cost and low immune responses, which are highly acceptable for clinical application and might provide different imaging and prognostic information compared to monoclonal antibodies.

In the present study, we observed that ATS markedly decreased the viability of HCT-15 cells under hypoxia condition. Previous studies indicated that sulfonamides could inhibit the activity of CA9 and resulted in pH regulation dysfunction in tumor cells [33, 34, 62]. Said and colleagues have shown that the specific chemical CA9 inhibitors, acetazolamide and SU.D2, are leading to an inhibition of CA9 in protein and mRNA levels during tumorgenesis of glioblastoma [63]. In addition, we also found that ATS bound to hypoxic HCT-15 cells via interacting with CA9. Therefore, we presume that the sulfonamide derivative decreases the CRC cell viability due to the insufficient activity and CA9 expression and thus they are unable to maintain the acidic tumor environment.

Hypoxia leads to stabilization and transcriptional activation of HIF-1α, which is a transcriptional factor for regulating hypoxia-inducible-genes expressions, including *CA9* gene [64]. A previous report exhibited a predominantly positive correlation between CA9 expressions and the levels of tumor hypoxia in cervical carcinoma [65]. In this study, we observed that the hypoxia level was remarkably enhanced in HCT-15 induced 4-week xenograft mice as compared with control. In addition, the CA9 expressions in tumors of HCT-15 induced 4-week xenograft mice were also higher than control group. Therefore, we believed that HCT-15 induced 4-week xenograft mice are desirable animal model for CA9 imaging. CA9 expressions are primarily associated with the areas of low pH and high rates of cell proliferation in normal tissue. In the human, CA9 levels are generally limited to a total exclusivity in the gastrointestinal tract epithelium, namely glandular gastric mucosa, epithelium of the gallbladder and cryptic enterocytes of duodenum, jejunum, and ileum [66]. In the adult mice, however, the highest levels of CA9 in the normal tissues are restricted to a few tissues, including stomach, colon, and brain [67]. In this study, the hypoxia distribution assay presented that stomach and colon revealed highly fluorescent signaling in the HCT-15 xenograft mice. However, the CA9 levels in stomach and colon were lower than other organs of HCT-15 xenograft mice. Therefore, we considered that the un-expectable fluorescent contaminants in the gastrointestinal tract might lead to the false signaling production under IVIS detection.

In conclusion, we demonstrated that CA9 is a target protein for CRC, and serum CA9 may be a potential tool for analyzing CA9 expressions in CRC. ATS can specifically bind to CA9-overexpressed CRC cells *in vitro*. To characterize *in vivo* imaging and assess the binding efficacy, ATS-DTPA-^111^In was performed in HCT-15 xenograft mice for measuring and analyzing CA9 imaging by using nanoSPECT/CT instrument. ATS-DTPA-^111^In displayed higher tumor binding efficacy in HCT-15-induced xenograft mice. Therefore, ATS-DTPA-^111^In may be an ideal nuclear imaging agent for CRC diagnosis. The CA9 bound sulfonamide-based nuclear imaging may has potential to improve the care and welfare of patients suspected of having CRC and could be used to monitor patients with a history of CRC after surgery or drug treatment.

## MATERIALS AND METHODS

### Acquisition of the tissues and sera of CRC

Clinical tissues and sera were acquired from Buddhist Tzu Chi General Hospital and Cheng Hsin General Hospital, Taiwan. The study protocol was approved by regulatory authorities and Institutional Review Boards of both institutions. Signed and informed written consent were obtained from CRC patients and healthy volunteers. The primary tumors (T) and adjacent non-tumor (NT) tissues from the individuals of CRC were captured followed by surgery. Tumor histopathology, grade, and stage were assigned according to the rules of American Joint Commission on Cancer Staging (AJCCS) system.

### Cell lines

Human colorectal carcinoma cells (HCT-15), human normal colorectal cells (FHC), rat liver normal cells (Clone 9), and human proximal tubule cells (HK2) were purchased from the American Type Culture Collection (ATCC) and maintained in ATCC's recommended culture medium with 10% of fetal bovine serum. All cells were incubated at 37°C and 5% CO_2_.

### Animals

The male BALB/c nude mice and male BALB/c littermates (7–8 weeks of age, weighing 21–23 g) were acquired from BioLASCO Taiwan Co., Ltd. These mice were selected and housed on a 12-h light cycle at 22°C and given food (mouse standard diet) or water *ad libitum*. All animal studies were approved by the institutive ethical review committee and were carried out in accordance with government regulations and NIH guidelines on the care and welfare of laboratory animals.

### Human serum CA9 assay

The serum CA9 levels in the clinical serum samples (50 colorectal cancer patients and 30 control participants) were analyzed by ELISA system (eBioscience, USA). Measurements were performed strictly according to the manufacturer's protocol and quality control was ensured. Samples were run in duplicate and serial dilution was performed in order to fall within the detection limits of the assay (0–300 pg/ml).

### Immunoblotting

Cells and tissues were lysed in the RIPA buffer (Sigma-Aldrich, USA) containing 50 mM Tris-HCl, pH 8.0, with 150 mM sodium chloride, 1.0% NP-40, 0.5% sodium deoxycholate, and 0.1% sodium dodecyl sulfate. Bradford protein Assay Reagent Kit (Bio-Rad, USA) was used to estimate protein concentration. The 10 μg of protein samples was diluted in 2X Laemmli sample buffer (Sigma-Aldrich, USA) containing 4% SDS, 20% glycerol, 10% 2-mercaptoethanol, 0.004% bromophenol blue and 0.125 M Tris HCl, pH approx. 6.8 and then separated by the 4–12% sodium dodecyl sulfate polyacrylamide gel electrophoresis, and then transferred onto the Immun-Blot® PVDF membranes (Bio-Rad, USA). These protein transferred-membranes were blocked in blocking buffer (Goal Bio, USA) for 2 min at room temperature. Membranes were incubated overnight at 4°C with primary antibodies for CA9 (#SAB5300133, Sigma-Aldrich), Ki-67 (#HPA030522, Sigma-Aldrich), PCNA (#HPA000451, Sigma-Aldrich), and then washed five times in Tris buffered saline with 0.1% tween-20 for 5 min. After washing steps, membranes were incubated with horseradish peroxidase-conjugated secondary antibody (diluted 1: 3000) for 1 hour at 4°C. Following the wash steps as described above, the proteins were analyzed by using ECL (Clarity™, Bio-Rad, USA) and monitored with a luminescent image analyzer (LAS-4000 mini, GE Healthcare, England). The immunoblotting bands were quantified by densitometric analysis using Multi Gauge v3.2 software.

### Histology and immunohistochemistry

The samples were sectioned at 10 μm with a cryostat (HM525, Microm, Germany), and sections were thaw-mounted on gelatin-coated glass slides. For histology staining, these 10-μm-thick tissue section slides were stained with hematoxylin and eosin for morphology detection. The methylene blue stain was performed to detect the cancerous lesions. For immunohistochemistry, immunostaining was performed by immunoperoxidase secondary detection system (Merck Millipore, Germany) and the manufacturer's instruction with a modification. Briefly, sections were incubated for 1 h at 4°C with blocking reagent. The primary antibodies for CA9 (#SAB5300133, Sigma-Aldrich) and HIF-1α (#H6536, Sigma-Aldrich) were used. After washing in PBS, the sections were incubated for 30 min at room temperature with a secondary antibody reagent. The sections were washed again in PBS and incubated for 15 min at room temperature with streptavidin HRP reagent. After a final wash in PBS, the sections were reacted with freshly prepared DAB Chromogen Reagent for 15 min. Sections were counterstained with hematoxylin counter stain solution (Sigma-Aldrich, USA) and coverslipped with Malinol (Muto Pure Chemicals, Tokyo, Japan). Images of the sections were obtained using a biological microscope Olympus DP70 (Olympus, Tokyo, Japan) and DP controller digital imaging software for research (Olympus) under 400 or 1000 magnification.

### Cell viability assay

WST-8 assay (Sigma-Aldrich, USA) was performed for cell viability measurement. Cells were seeded in 96-well plates at a density of 1 × 10^4^ cells/well and cultured in 37°C and 5% CO_2_ for 24 hours. After pre-incubation, each well, which containing about 80% confluent cells were treated with ATS (1–500 μg/ml) for 24 and 48 hours under normoxic and hypoxic condition and then 10 μl of WST-8 solution was added to each well. After 2 hours-incubation at 37°C, the samples were analyzed by using a microplate reader (Bio-Rad, USA).

### Flow cytometry analysis

HCT-15 and Clone 9 cells were seeded at 2 × 10^6^ cells/ml in 10 cm dish, which were incubated under normoxic or hypoxic condition for 24 hours. In competitive group, HCT-15 cells were pre-treated with ATS (10 μg/ml) for 1 hour and incubated in hypoxic condition. After 24-hour incubation, medium was removed and washed with PBS for three times, and then incubated with FITC-labeled ATS (ATS-FITC, 10 μg/ml) for 3 hours. Cells were washed and harvested for flow cytometric analysis by FACSCalibur Flow Cytometer (BD Bioscience, USA).

### Immunofluorescence staining

The ATS-FITC binding assay was analyzed at HCT-15 cells by immunofluorescence staining. Briefly, cells were seeded in 8-well Millicell EZ slide (Merck Millipore, Germany) with cultured medium for 16 hours at 37°C and 5% CO_2_. Then, each well (2 × 10^3^ cells) was incubated 37°C and 5% CO_2_ for 24 hours under normoxic and hypoxic condition. ATS (10 μg/ml) was a competitor for competitive inhibition assay. Then, cells were washed by PBS and then fixed using 4% paraformaldehyde for 15 min at room temperature. Fixed-cells were incubated with SuperBlock Blocking Buffers (ThermoFisher Scientific, USA) for 1 hour at room temperature. After blocking reaction, cells were incubated with fluorescent labeled-ATS (10 μg/ml) for 4 hours at room temperature. DAPI was used to stain the cell nuclei at a concentration of 0.2 μg/ml for 10 minutes. After cells washing, the images were digitally acquired with a fluorescence microscope (BX53, Olympus, Japan), coupled to a CCD camera. Digital images were taken using Image-Pro Plus software (Media Cybernetics, USA). Each staining was repeated 3 times in 3 different cultures.

### ATS and CA9 interaction assay

To assess the interaction between CA9 and ATS in HCT-15 cells, co-immunoprecipitation (co-IP) experiment was performed. Briefly, hypoxia for 24 and 48 hours, HCT-15 and FHC cells (1 × 10^6^ cells) were pre-treated with fluorescent labeled ATS (10 μg/ml) for 4 hours at 4°C. Then, the cells were harvested and lysed on ice for 30 minutes with lysis buffer, containing 150 mM NaCl, 0.5% sodium deoxycholate, 0.1% TX-100, 50 mM Tris, pH 8.0, and protease inhibitors cocktail. Cell debris was removed by centrifugation at 14,000 rpm for 10 min at 4°C. The supernatant of lysates was incubated with or without biotinylated-CA9 polyclonal antibody (2 μg/ml, Novus Biologicals, USA) in the presence of streptavidin agarose beads (Sigma-Aldrich, USA) at 4°C overnight. The immunoprecipitates were washed 5 times with washing buffer (150 mM NaCl, 20 mM Tris, pH7.4, 0.1% TX-100) and detected the fluorescent signaling by using a ELISA reader (Wallac 1420 VICTOR2™, Perkin Elmer, USA).

### HCT-15 xenograft mouse model and *in vivo* hypoxia imaging and CA9 distribution assay

HCT-15 cells (3 × 10^6^) were subcutaneous inoculated into the right flank of nude mice (*n* = 5 per each group). Tumors were established for one week and four weeks. For *in vivo* hypoxic imaging assay, the hypoxia-detected agent labeled with fluorescent (HypoxiSense 680, PerkinElmer, USA) was used. The commercial hypoxia-detection agent is a CA9 specific fluorescent *in vivo* imaging agent that can be used to image CA9 overexpression in tumors in response to regional tumor hypoxia. The agent was intravenously injected into tail vein of HCT-15 xenograft mice. After injection for 24 hours, *in vivo* imaging system (IVIS, PerkinElmer, USA), a fluorescent detection system, were performed to capture the whole animal body images for analyzing hypoxic distribution.

### DTPA labeled-sulfonamide synthesis and measurement

For preparation of diethylene triamine pentaacetic acid (DTPA) labeled-sulfonamide (ATS-DTPA), ATS and DTPA dianhydride (w/w 1:50) was incubated at 25°C with bicarbonate buffer (pH 8.5) for 4 hours. Then, the conjugated-compounds were purified by high pressure liquid chromatography with G-10 column. For molecular weight assay, liquid chromatography-mass spectrometry (LTQ Orbitrap XL™ Hybrid Ion Trap-Orbitrap, Thermo Scientific, USA) was performed for ATS analysis. And matrix-assisted laser desorption/ionization time-of-flight mass spectrometry (UltraflexIII TOF/TOF, Bruker Daltonics GmbH, Germany) was performed for DTPA and ATS-DTPA detection. Briefly, the compounds were crystallized with an equal volume of freshly prepared α-cyano-4-hydroxycinnamic acid matrix (10 mg/ml in 50% acetonitrile/0.5% TFA) for mass spectrometry analysis. The calibration standards were used as external calibrator with mass accuracy within 100 ppm. The spectra were processed using FlexAnalysis™ 3.0 software (Bruker, Germany). The ATS-DTPA conjugation efficiency with indium-111 was evaluated from the radiolabeling yields of ATS-DTPA-^111^In determined by instant thin layer chromatography (ITLC) (AR-2000 radio-TLC Imaging Scanner, Bioscan, France) on silica gel impregnated glass fiber sheets (PALL corporation, USA) using PBS buffer, pH 7.4, as the mobile phase.

### The stability assay of ATS-DTPA and ATS-DTPA-^111^ In *in vitro*

To investigate the stability of ATS-DTPA, the levels of ATS-DTPA were measured to observe the variation of molecular weight based on pH of the solvent. Briefly, the freeze-dried ATS-DTPA was dissolved in aqueous solvent with different pH: 5.5, 7.4, and 8.5 at 37°C. Then, the alteration of ATS-DTPA levels in an aqueous solution over time (up to 4 days) was estimated. Samples (200 μl) were collected from the stock solution and analyzed by using HPLC. For ATS-DTPA-^111^ In stability assay, the radiolabeled-compounds were incubated in human and bovine serum for 144 hour. Then, the radiolabeling yields of ATS-DTPA-^111^In were analyzed by ITLC every 24 hour. Each assay was repeated 3 times in 3 different containers.

### *In vivo* CA9 nuclear imaging

*In vivo* CA9 nuclear imaging was measured on male BALB/c nude mice, subcutaneous inoculated with HCT-15 cells (2 × 10^6^ cells). Tumors were established for four weeks. For CA9 nuclear imaging assay, all mice received indium-111 labeled with or without ATS conjugation via intravenous injection with 2 mCi of indium-111. NanoSPECT/CT (Mediso Medical Imaging Systems, USA) was performed to capture the imaging for detecting CA9 distribution at 6 and 24 hours. The radioactive intensity was estimated against the baseline (i.e., the radioactive intensity of muscle derived from a leg without the tumor embedded).

### *Ex vivo* bio-distribution study

For tissue bio-distribution assay, 2 × 10^6^ HCT-15 cells were injected subcutaneously into the right hind limb. Tumor size and the state of health of animals were checked regularly. Approximately 4 weeks after inoculation, all animals were performed isofluorane anesthesia. Then, mice received a single tail vein injection of ATS-DTPA-^111^In or DTPA-^111^In (0.3–0.4 MBq [8–11 μCi]). At 24 hours post-injection, mice were sacrificed and then removed the organs and blotted dry. All samples were weighed and the radioactivity was analyzed using a gamma counter (1470 WIZARD, PerkinElmer, USA) along with known standards. Data points were corrected for radioactive decay. The percent injected dose per gram of tissue (%ID/g) was calculated.

### Toxicity evaluation for ATS-DTPA

Forty male BALB/c mice were divided into 4 groups, each consisting with 10 animals. Group I was control group and received only vehicle (PBS). Group II, III, and IV were treated with ATS-DTPA at doses of 0.5, 5, 50 mg/kg, respectively. All groups were intravenously administrated with a signal dose. The body weight changes % of all group were determined daily for 14 days. Fourteen days after injection, blood was collected from retro-orbital sinus of anesthetized mice and serum was separated for determination of blood urea nitrogen (BUN), serum creatinine, alanine aminotransferase (ALT), and aspartate aminotransferase (AST). Mice were sacrificed and the kidneys and livers were isolated. The kidneys and livers were immersed in buffered 4% paraformaldehyde solution for 48 h to obtain histological sections.

### Statistical analysis

All data analysis was performed by ANOVA followed by post hoc analysis with Bonferroni's test. Data were presented as means ± SEM. Differences between means were considered signification when *p* value < 0.05. Statistical analysis was performed by using GraphPad Prism V5.01 software (GraphPad Software, Inc. USA).
